# Unstructured road extraction and roadside fruit recognition in grape orchards based on a synchronous detection algorithm

**DOI:** 10.3389/fpls.2023.1103276

**Published:** 2023-06-02

**Authors:** Xinzhao Zhou, Xiangjun Zou, Wei Tang, Zhiwei Yan, Hewei Meng, Xiwen Luo

**Affiliations:** ^1^ College of Mechanical and Electrical Engineering, Shihezi University, Shihezi, China; ^2^ Foshan-Zhongke Innovation Research Institute of Intelligent Agriculture, Foshan, China; ^3^ Foshan Sino-tech Industrial Technology Research Institute, Foshan, China; ^4^ College of Engineering, South China Agricultural University, Guangzhou, China; ^5^ Guangdong Provincial Key Laboratory of Agricultural Artificial Intelligence (GDKL-AAI), Guangzhou, China

**Keywords:** non-structural environment, machine vision, fruit harvesting robot, deep learning, roadside fruits detection

## Abstract

Accurate road extraction and recognition of roadside fruit in complex orchard environments are essential prerequisites for robotic fruit picking and walking behavioral decisions. In this study, a novel algorithm was proposed for unstructured road extraction and roadside fruit synchronous recognition, with wine grapes and nonstructural orchards as research objects. Initially, a preprocessing method tailored to field orchards was proposed to reduce the interference of adverse factors in the operating environment. The preprocessing method contained 4 parts: interception of regions of interest, bilateral filter, logarithmic space transformation and image enhancement based on the MSRCR algorithm. Subsequently, the analysis of the enhanced image enabled the optimization of the gray factor, and a road region extraction method based on dual-space fusion was proposed by color channel enhancement and gray factor optimization. Furthermore, the YOLO model suitable for grape cluster recognition in the wild environment was selected, and its parameters were optimized to enhance the recognition performance of the model for randomly distributed grapes. Finally, a fusion recognition framework was innovatively established, wherein the road extraction result was taken as input, and the optimized parameter YOLO model was utilized to identify roadside fruits, thus realizing synchronous road extraction and roadside fruit detection. Experimental results demonstrated that the proposed method based on the pretreatment could reduce the impact of interfering factors in complex orchard environments and enhance the quality of road extraction. Using the optimized YOLOv7 model, the precision, recall, mAP, and F1-score for roadside fruit cluster detection were 88.9%, 89.7%, 93.4%, and 89.3%, respectively, all of which were higher than those of the YOLOv5 model and were more suitable for roadside grape recognition. Compared to the identification results obtained by the grape detection algorithm alone, the proposed synchronous algorithm increased the number of fruit identifications by 23.84% and the detection speed by 14.33%. This research enhanced the perception ability of robots and provided a solid support for behavioral decision systems.

## 1 Introduction

Around the world, fruit plays an increasingly vital role in agriculture and economy. According to Food and Agriculture Organization of the United Nations (FAO), the total value of grape production has increased steadily since 1991, to more than $80 billion by 2020. Fruit harvesting is characterized by having limited work cycles and being labor intensive and time-consuming. With aging of the population and lack of rural labor force, labor costs have increased year by year (Wu et al., 2021; Li Y. J. et al., 2022). Under the influence of the COVID-19 pandemic and related policies (Aamir et al., 2021; Nawaz et al., 2021; Bhatti et al., 2022a; Bhatti et al., 2022b), the contradiction between labor demand and labor costs has become more prominent (Liang et al., 2021; Lin et al., 2022). This has had a negative impact on traditional hand-picking operations. With the deterioration of environmental issues (Bhatti et al., 2022; Galvan et al., 2022; Tang et al., 2023a), all the above factors pose a great challenge to China’s fruit industry. With the rapid development of modern information technology and artificial intelligence technology, fruit harvesting robots and their related technologies have attracted extensive attention (Chen M. et al., 2020; Fu L. H. et al., 2020; Fu L. et al., 2020; Rysz and Mehta, 2021; Yang, 2021; Kang et al., 2022; Wang X. et al., 2022; Wu Z. et al., 2022).

As the basis of autonomous navigation, road detection is crucial to the precise operation of fruit harvesting robots and has become the focus of research in recent years (Ma et al., 2021; Sun et al., 2022). The main objective of road extraction is to extract the road regions from the background in a complex scene to lay the foundation for determining the navigation path. According to the characteristics of roads, they can be divided into two categories: structured roads and unstructured roads. Structured roads are standardized roads similar to urban roads and expressways, with clear lane markings, regular road edges, and distinct geometric features. Unstructured roads are those with irregular road edges, unclear road boundaries, no lane lines, and similar to orchards and rural areas. Compared to structured roads, unstructured roads have a more complex environmental background. For the most part, the surface of the unstructured road is mostly uneven, with a few random weeds. In contrast, the problem of unstructured road extraction is more complicated.

Research of road detection is usually divided into machine learning segmentation methods and traditional algorithms based on image features.

Road segmentation methods of machine learning are mainly divided into clustering (Zhang Z. Q. et al., 2022b), seed support vector machine (SVM; Liu et al., 2018), deep learning (Li et al., 2020), and other methods. Yang Z. et al. (2022) have proposed a visual navigation path extraction method based on neural network and pixel scanning. They introduced Segnet and Unet networks to improve the segmentation effect of orchard road condition information and background environment and adopted sliding filtering algorithm, a scanning method, and a weighted average method to fit the final navigation path. Lei et al. (2021) have combined improved SVM and two-dimensional lidar point cloud data to detect and identify unstructured roads. Wang E. et al. (2019) have realized road extraction of complex scenes by combining illumination invariant images and analyzing probability map and gradient information. Kim et al. (2020) have implemented automatic path detection in semi-structured orchards based on patch and CNN neural network methods. Alam et al. (2021) have implemented road extraction in structured and unstructured environments by combining multi-nearest neighbor classification and soft voting aggregation. Some scholars have also studied methods for road extraction in remote sensing based on machine learning methods (Xin et al., 2019; Chen et al., 2022; Guan et al., 2022; Yang M. et al., 2022). However, relevant research has been more on the basis of urban development analysis or traffic network monitoring and other fields, which are not applicable to picking robots. Machine learning usually does not require manual feature selection. However, this method requires specific network training and a large number of training sets and has certain limitations.

In the method based on image-feature analysis, some scholars use color, texture and other features to distinguish road and nonroad areas by establishing models and other methods. Zhou et al. (2021) have used the H component to extract the target path for the sky region. Chen J et al. (2020; 2021) have used an improved gray scale factor and the maximum interclass variance method (Otsu) method to extract gray scale images of soil and plants and realized segmentation of soil and plants in the greenhouse environment. Qi et al. (2019) have segmented the road region based on a graph-based manifold ranking approach and used binomial functions to fit the road region model, thus realizing road recognition in rural environment. Some scholars have also considered the vanishing point and other spatial structure features in the process of road extraction. Su et al. (2019) have adopted the Dijkstra method combined with single-line lidar to realize road extraction on the basis of the constraints of pre-vanishing points of illumination-invariant images. Phung et al. (2016) have realized pedestrian lane detection based on an improved vanishing point estimation method combined with geometry and color features. However, the detection of vanishing points is time-consuming and mostly applied to structured road detection (Xu et al., 2018), which is not suitable for dealing with unstructured roads.

To realize autonomous walking and precise operation of fruit harvesting robots in orchard environments and aiming at the uncertainty of random distribution of roadside fruit and road complexity, it is necessary to deeply study the problem of synchronous road extraction and fruit identification. This study enables robot perception of barrier-free road areas and roadside fruit distribution in the current environment and can provide an inferential basis for robot global operational behavior decisions in complex orchard environments. Moreover, this study can lay the foundation for the joint control and operation of navigation and picking based on visual guidance in the panoramic environment of wild orchards. However, current approaches have only focused on road extraction, without considering the roadside fruit detection. In this case, the autonomous decision-making function of the robot cannot perform reasonable picking responses and navigation path planning based on the random distribution of fruits along the road, which is detrimental to the intelligent global continuous operation of the robot.

In terms of object detection, neural networks have been widely used in the field of smart agriculture (Khaki and Wang, 2019; Tang et al., 2020; Feng et al., 2022; Fu et al., 2022), and You Only Look Once (YOLO), as one of the fastest target detection models at present, has also been rapidly developed (Ye et al., 2020; Ning et al., 2022; Wang X. Y. et al., 2022). For example, due to the excellent performance of the YOLOv5 model in terms of accuracy and running time, it has been greatly valued by scholars in the research of crop growth-morphology recognition (Lv et al., 2022; Rong et al., 2022; Wu F. et al., 2022), detection and positioning (Fang et al., 2022; Jintasuttisak et al., 2022; Li G. et al., 2022; Wang H. et al., 2022), tracking counting (Lyu et al., 2022; She et al., 2022; Zang et al., 2022), and pest recognition (Li S. et al., 2022; Qi et al., 2022; Zhang et al., 2022).

Given the importance of detecting and locating fruit for picking robots, researchers have explored various fruit detection and location methods based on neural networks (Wang C. et al., 2019; Ge et al., 2022; Jia et al., 2022; Zhou et al., 2022; Tang et al., 2023c). To improve the operational efficiency and success rate of picking robots, researchers have gradually shifted their focus to picking-path planning algorithms and picking decision systems based on fruit detection (Lin et al., 2021; Wang Y. et al., 2022). For example, Xu et al. (2022) have proposed an efficient combined multipoint picking scheme for tea buds through a greedy algorithm and ant colony algorithm, which improved picking efficiency and overall picking success rate. Ning et al. (2022) proposed a method for recognition and planning robotic picking sequences for sweet peppers based on an improved YOLOV4 model and a principle of anticollision picking within picking clusters. The method can accurately detects sweet peppers, reduces collision damage, and improves picking efficiency in high-density orchard environments. Rong et al. (2022) have proposed an obstacle avoidance method that combines end-effector grasping-pose adjustment and harvesting sequence planning based on a custom manipulator. Experiments show that the method significantly reduced the impact of collision on the picking and improved the success rate of tomato picking. Although some progress has been made in the study of local target detection and picking planning, there have been few reports on the synchronization information perception needs of picking robots to autonomously pick and walk.

To implement the behavioral decision-making function of the picker robot to walk autonomously and pick accurately throughout the entire process in a large-area orchard environment, road extraction and roadside fruit identification should first be implemented in the current working scenario. Currently, many algorithms only focus on road extraction and ignore the fruit distribution along the road, which leads to the serious problem that picking robots are not robust enough to adapt to the changing orchard environments. Therefore, a road extraction and roadside fruit synchronous recognition algorithm based on unstructured road was proposed in this study. The main contributions of this study were as follows:

(1) Currently, numerous studies have focused on extracting unstructured roads without considering the synchronous recognition of roadside fruits, which is detrimental to improving the ability of picking robots to obtain environmental information. Motivated by the need for cooperative behavioral decision-making in fruit picking robots, this study proposed a framework for unstructured road extraction and synchronous recognition of roadside fruit. This framework can effectively improve the ability of fruit-picking robots to extract crucial information from the picking environment and lay a foundation for multitask parallel processing, thereby enabling cooperative behavioral decision-making among fruit-picking robots.
(2) Due to the randomness and complexity of orchard environments, the results of road extraction directly from raw images were not very accurate and contained a large number of misidentified regions. An image preprocessing method based on image enhancement and filtering preprocessing was designed here which reduced the influence of interference existing in the complex orchard environment. Simultaneously, this approach enhanced the precision of road extraction results and was of great importance for improving the quality of road extraction.
(3) The irregular road edges of unstructured roads and various interference factors in orchards considerably impacted the stability of the road extraction results. To address this issue, analyses of orchard images were conducted to optimize the gray factor and enhance its adaptability to field orchards. A two-space fusion unstructured road extraction algorithm was proposed, which used color channel enhancement and gray factor optimization and demonstrated great adaptability to interference factors, such as shadow, uneven lighting, grapevine on the side of the road, and strong contrast between light and shade in the field complex environment.
(4) A fusion algorithm based on the road extraction algorithm and roadside fruit detection algorithm was constructed. Based on the detection requirements for roadside grapes in wide-field environments, YOLO models were compared, selected, and optimized for their parameters. Subsequently, the three functions of image preprocessing, road extraction, and roadside grape recognition were integrated to construct a synchronous recognition algorithm, allowing for the simultaneous extraction of road and other key information during the fruit-picking process. The proposed algorithm provided information for decision-making and reasoning of collaborative behavior of key parts of the robot, so as to improve robot adaptability to randomly distributed fruit.

This study will lay a foundation for the construction of robot behavior decision control system, and it is of great significance for improving the intelligence, accuracy, and stability of robot field autonomous work.

The rest of this report is organized as follows. Section 2 introduced the materials and data. Section 3 explained the structure and implementation of the algorithm. Section 4 presented the experimental results and comparative discussion. Finally, Section 5 summarized the study and plans for future work.

## 2 Materials and data acquisition

### 2.1 Experimental platform for wine grape picking and moving

**Figure 1:**
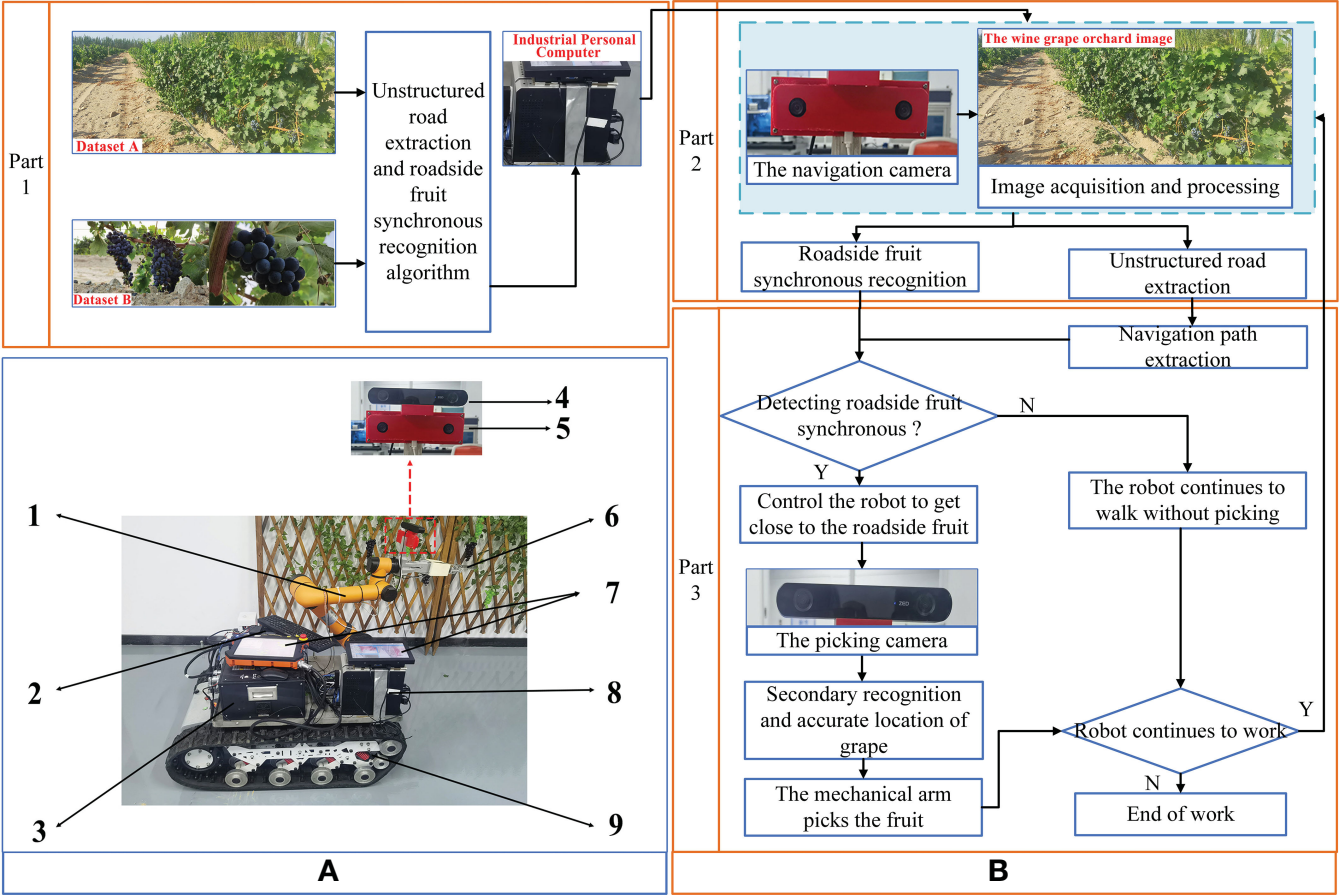
Overall layout and control flow of test platform. **(A)** Overall layout of test platform. Mechanical arm (AUBO-i5, AUBO), 1; Battery, 2; Controller, 3; Camera for picking (HBV-1714, Huiber Vision Technology Co., Ltd), 4; Camera for navigation (ZED 2, Stereolabs), 5; End-effector, 6; Human Machine Interaction, 7; Industrial Personal Computer (IPC), 8; and Track car, 9. **(B)** Control flow of the test platform.

This study was based on the wine grape visual mobile picking robot that was independently designed and developed. The overall layout of the test platform is shown in **Figure 1A**. The test platform was battery powered to operate in the orchard. The length and width of the platform were 1.065 and 0.7 m, respectively, and the maximum climbing capacity was 30°. Two cameras were installed on the end-effector of the platform as picking camera and navigation camera, separately.

The control process of the experimental platform was divided into three main parts (**Figure 1B**). The first part of the control system was to construct algorithms for unstructured road extraction and roadside fruit synchronization recognition based on the collected datasets A and B. Then, the industrial personal computer (IPC) implemented the algorithm-based key information acquisition, recognition, and behavioral decisions. The second part of the control system was to use the IPC to control the navigation camera for orchard road extraction and roadside fruit recognition. By recognizing the distinction between unstructured roads and chaotic backgrounds, as well as the classification and recognition of roadside grapes and grapevines, it provided a judgment basis for the IPC to distinguish the presence of roadside fruit and lay the foundation for behavioral decisions. Based on the above information, the third part of the control system extracted the navigation path of the orchard and judged the presence of fruit in the current roadside area. If there were fruit on the roadside, the controller controlled the tracked vehicle to approach the fruit area of the roadside fruit tree and fed the information to the robotic arm and another set of stereo camera for precise positioning (the picking camera for short) for picking operations. Using the picking camera, fruit could be re-identified and accurately positioned to achieve fruit picking in complex environments. The work of this study mainly implemented the first part of the control system.

### 2.2 Experimental subjects

Wine grapes and non-structural orchards were taken as experiment subjects in this research. Wine grape fruit are clustered in shape and usually purple at maturity, with a clear color difference from leaves. The planting mode is usually in rows with a certain row spacing. As the fruit distribution and planting patterns of wine grapes are similar to other row-grown crops, such as tomato and dragon fruit, the results of this study are expected to be extendable to other types of fruit.

### 2.3 Image acquisition

In August 2022, experimental images were obtained from Xinyu Winehouse (Bohu County, Bazhou, Xinjiang). The device used for dataset sampling was an OPPO R11 mobile phone with a 20-megapixel rear camera. All images were taken under natural daylight conditions without artificial light sources and saved in Joint Photographic Expert Group (jpg) format with image size 4608×2128 pixels.

**Figure 2:**
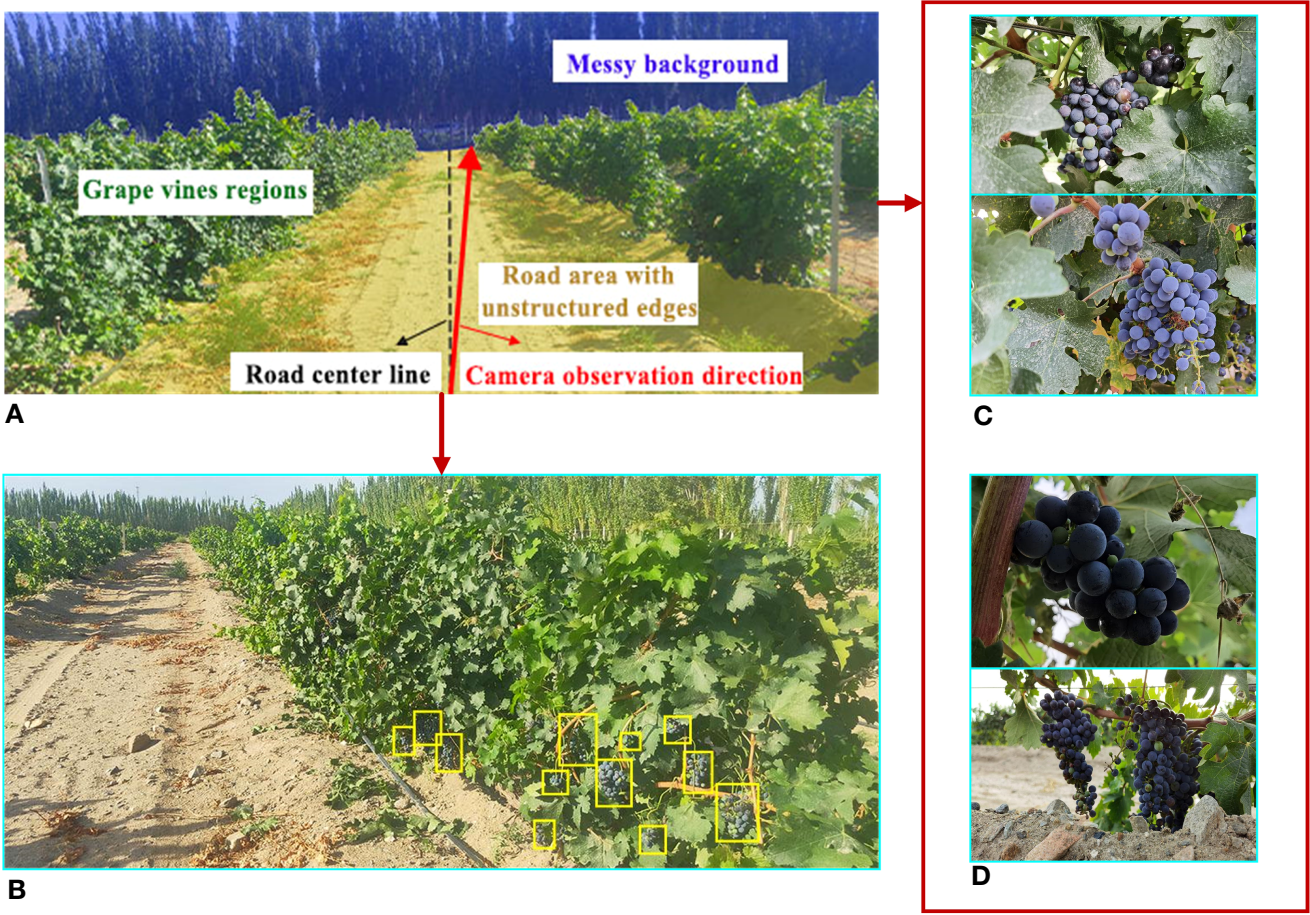
Schematic diagram of the acquisition process of test images. **(A)** The camera observation direction. **(B)** Example image of the wine vineyard. **(C)** Examples of frontlight images. **(D)** Examples of backlight images.

The collected images were divided into datasets A and B. The original images of vineyards in dataset A included roads and vines. As the algorithm proposed in this study was intended to provide a basis for behavioral decisions of grape-picking robots, the focus was on the region of unstructured road and distribution of fruit in a unilateral grape row. Therefore, during the collection process of dataset A, the camera observation direction was biased to the right of the road center line (**Figure 2A**). A total of 337 typical orchard images were selected, in which the roads in the grape orchard environment had features of shadow and irregular road edges (**Figure 2B**). Dataset B was composed of 1081 valid images showing wine grape clusters, including grape samples in numerous cases, with images of grapes in front and backlight (**Figures 2C, D**).

### 2.4 Image datasets

To simulate the vision system of the picking robot, valid grape and orchard image samples were collected under different conditions of illumination, weather, sampling distance, and differing severity of fruit adhesion and occlusion, forming datasets A and B.

**Figure 3:**
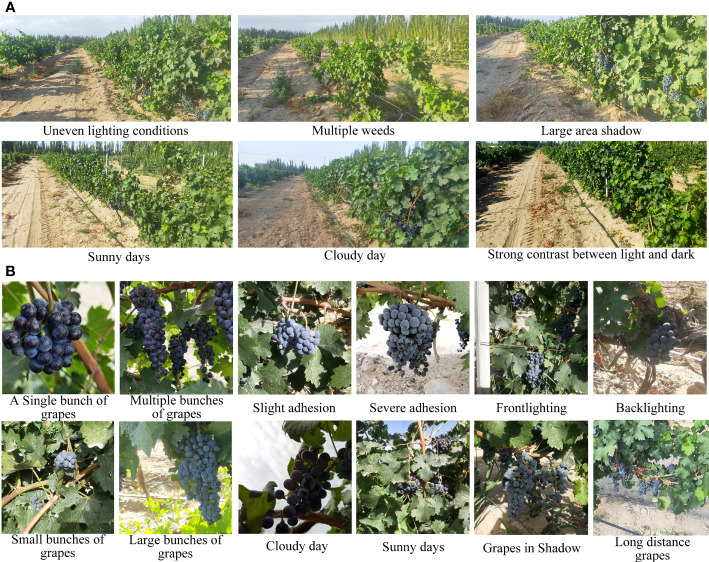
Natural images of vineyards and wine grape clusters. **(A)** Natural images of vineyards. **(B)** Natural images of wine grape clusters.

Dataset A consisted mainly of orchard images with uneven lighting, with multiple weeds, with large shadows, in different weather conditions, and with different light and shade contrasts (**Figure 3A**).

The natural images of grapes (dataset B) mainly included images of single cluster grape, multiple clusters grape, slightly-adhered grape, severely-adhered grapes, front and back illumination, small string grapes, large cluster grapes, and grapes on a sunny day, on a cloudy day, and in shadow as well as grapes at different sampling distances. Their representative images are shown in **Figure 3B**.

Datasets A and B were challenging considering the effects of complex background, light levels, shadows, randomly distributed fruits, weeds, and different levels of fruit occlusion. Images of grapes and vineyards in a typical complex environment were contained in dataset A and B (**Figure 3**).

Dataset A was only employed for testing the performance of unstructured road extraction and the overall algorithm, with 100 images in this dataset randomly selected as the test set for algorithms in this study. To improve algorithm efficiency, the processing image size of the algorithm was set to 1024×473 pixels.

Dataset B was used for training and validation of the fruit model on the YOLOv7 roadside. Under LabelImg (https://github.com/tzutalin/labelImg), grapes in images were manually annotated as rectangles with the label “fruit,” which then saved annotation files in “txt” format. Among them, the whole image set was randomly divided into training and validation sets with a ratio of 9 to 1.

## 3 Methodology and algorithm description

**Figure 4:**
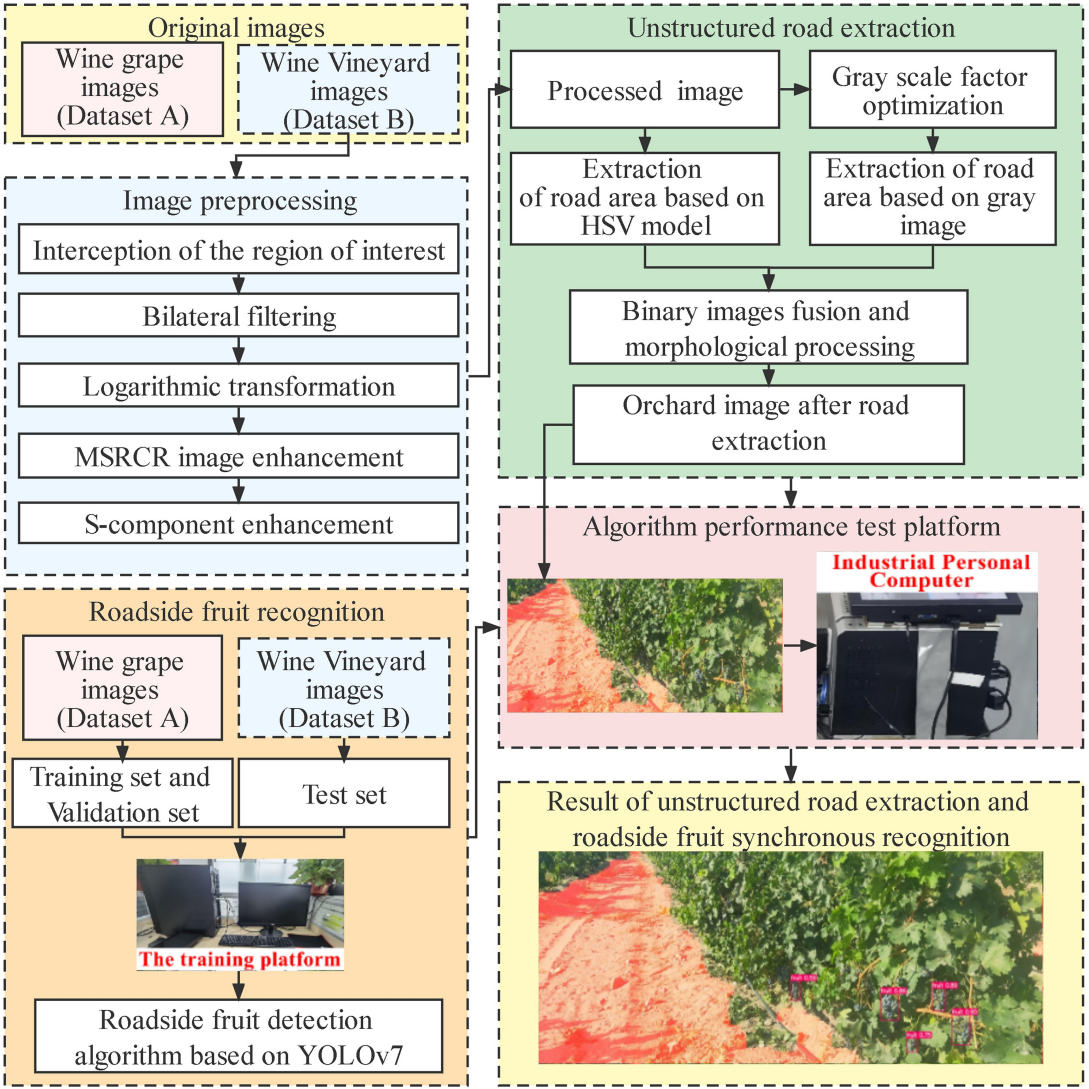
Flow chart of the entire algorithm.

In this study, the algorithm content was mainly divided into two parts: First, the road in the unstructured orchard environment was extracted. Second, taking the road extraction results as input, roadside fruit were identified through YOLOv7 to realize the synchronous information extraction of the road extraction and roadside fruit detection. The algorithm process of this study is shown in **Figure 4**.

### 3.1 Image preprocessing

**Figure 5:**
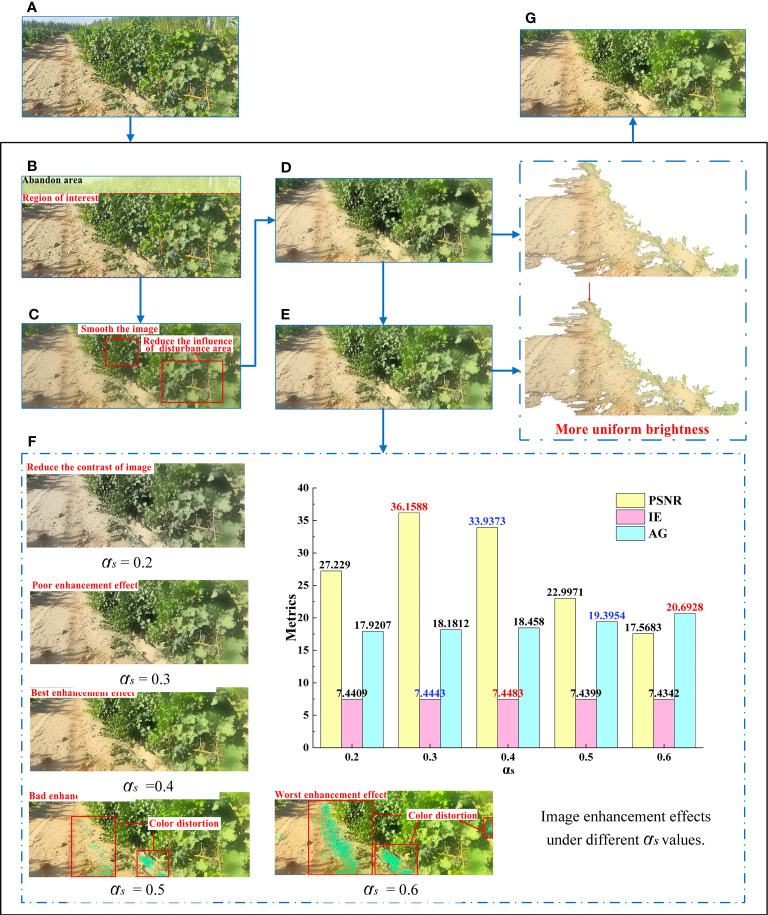
Process and results of image preprocessing algorithm. **(A)** Original image. **(B)** Region of interest. **(C)** Bilateral filtering result. **(D)** Log space transformation. **(E)** Image enhancement based on the MSRCR algorithm. **(F)** S-component enhancement. **(G)** Results of image preprocessing algorithm.

During image acquisition in the orchard, it was inevitable to be disturbed by external environmental noise, such as uneven light and dust, which made the image details unclear and led to road extraction errors. Therefore, this study preprocessed the images in dataset A, which was of great significance for improving the quality of road segmentation (Wang et al., 2018; Zhang P. et al., 2022). The image preprocessing method proposed in this study consisted of five steps, with the processing procedure and image quality enhancement results illustrated in **Figure 5**. Further details can be found in Sections 3.3.1 - 3.1.5.

#### 3.1.1 Interception of regions of interest

The images in dataset A were composed of sky, road, grapes, and messy background, among which the sky and messy background were mainly distributed at the top of an image. In the image processing process, if the entire image captured by the camera was merely taken as the research object, a substantial amount of computation would be required and a significant amount of interference inevitably occurs, which will reduce road extraction accuracy. To this end, only the regions of interest (ROI) of the image was extracted for subsequent processing. After a number of experiments, it was found that the appropriate ROI was at the lower 5/6 position of the image (**Figure 5B**). This ROI selection not only significantly reduced the calculation volume, but also ensured the accuracy and reliability of unstructured road extraction.

#### 3.1.2 Bilateral filter

A bilateral filter can smooth the image while maintaining edge details (Routray et al., 2020). To enhance and improve contrast between the foreground and background of the road to facilitate subsequent segmentation, a bilateral filter was used to process the present images. To reduce the influence of minor areas, such as vines, fruits, vine gaps, and cavities in subsequent segmentation, the key parameters of the bilateral filter (Liu et al., 2017) in this study were set to: diameter *d* of the pixel domain was 60, standard deviation of spatial domain 120, and standard deviation of intensity domain 60 (**Figure 5C**).

#### 3.1.3 Logarithmic space transformation

To enhance the details in the shadowed regions and provide images with enhanced details and uniform brightness for subsequent MSRCR processing, a logarithmic transformation of the V-component in hue, saturation, and value (HSV) space was used here to expand the low gray values and compress high gray values in this channel (**Figure 5D**). The standard form was


(1) S=c∗log(1+L)

where *S* is the correction image, *L* the source image, and *c* the gain adjustment parameter, which was set to 1.

#### 3.1.4 Image enhancement based on the MSRCR algorithm

After the above processing and observing the image under RGB color space, the altering influence of illumination was found not to be entirely eliminated. Therefore, the MSRCR algorithm was selected for image correction and enhancement here to obtain realistic images with reduced illumination effects. The resulting Equations 2–4 were expressed as:


(2) $$R_{MSRCR}(x,y)=C_{i}(x,y)R_{MSR}(x,y)$$

(3) $$R_{MSR}(x,y)=\sum_{1}^{N}\varphi_{n}\left\{\log I_{i}(x,y)-\sum_{1}^{N}\log\left[F(x,y)*I_{i}(x,y)\right]\right\}$$

(4) $$C_{i}(x,y)=\beta \left\{\log\left[\alpha I_{i}(x,y)\right]-\log\left[\sum_{1}^{N}\left(I_{i}(x,y)\right)\right]\right\}$$

The optimal functional form of MSRCR is shown in Equation 5, expressed as:


(5) $$\begin{array}{c} R_{MSRCR}(x,y)=\\ G\left\{C_{i}(x,y)\left[\log I_{i}(x,y)-\sum_{1}^{N}\log(I_{i}(x,y)*F(x,y)\right]+b\right\} \end{array}$$

where $I_{i}(x,y)$ is the color component image corresponding to each color channel, F(x,y) the Gaussian filter function, and $C_{i}(x,y)$ the color restoration factor of the *i* ^th^ color channel, $\varphi_{n}$ the weight, and *N* the number of spectral channel, where $\sum_{1}^{N}\varphi_{n}=1$ , *β* a gain constant, and *α* the strength of nonlinearity, *G* and *b* the final gain and offset values, respectively. The parameters of MSRCR in this study were configured according to the reference (Jobson et al., 1997).

#### 3.1.5 S-component enhancement

To enrich color information, this study adjusted the saturation channel S to enhance image quality, with the formulas described by Equations 6–7 (Huang et al., 2022), expressed as:


(6) $$S_{opt}=\alpha_{s}*T*S_{o\text{ri}}$$

(7) $$T=\frac{mean(R,G,B)+Max(R,G,B)+Min(R,G,B)}{mean(R,G,B)}$$

where $S_{opt}$ represents the enhanced saturation channel, $S_{o\text{ri}}$ the original saturation channel of S, mean(R,G,B) , Max(R,G,B) , and Min(R,G,B) the average, maximum, and minimum values of pixels corresponding to R, G, and B color channels, respectively, and *α_s_* and *T* the gain coefficients of the saturation channel, which control the enhancement degree of S channel image.

Qualitative and quantitative evaluation is significant for the evaluation of image quality. In the qualitative evaluation, the quality of the enhanced image was evaluated in color, contrast, and detail. By comparing the gain effect at different values, it was observed that, if the value of *α_s_* was too high or low, the image contrast was reduced or saturation too strong, which affected the visual effect of the image. When *α_s_* = 0.2, the contrast of the image was low, resulting in poor overall visual effect. When *α_s_* was greater than 0.5, there was significant color distortion despite the high contrast of images, resulting in partial loss of detail in the image. When *α_s_* = 0.3, although the tone of the image was better maintained, the enhancement effect was not obvious compared with the image without S-component enhancement. When *α_s_* = 0.4, the contrast of the image was improved significantly without obvious color distortion and the visual effect was the best.

In the quantitative evaluation, this paper evaluated the performance the processing results by three metrics Peak signal-to-noise ratio (PSNR, He et al., 2015), information entropy value (IE, Wang et al., 2021) and average gradient (AG, Zhang X. et al., 2022). PSNR has been widely used for measuring attributes like texture details enhancement, details preservation and contrast enhancement. A higher PSNR generally indicates that the processed image is of higher quality (Gupta and Tiwari, 2019). IE is mainly an objective evaluation index that measures how much information an image contains. The enormous IE value indicates that the enhancement image contains more image information. AG represents the degree of change in the gray value of the image, and is one of the criteria for judging the processing of image details and clarity. The large AG value indicates that the enhancement image contains more gradient information and detailed texture. The image enhancement quality evaluation parameters under different values of *α_s_* were shown in **Figure 5F**, where the optimal parameter values were marked in red and the second highest parameter values were highlighted in blue. Figure illustrated that the value of AG increased as the value of *α_s_* increased, indicating that the sharpness of the image was also enhanced progressively. However, color distortion occurred when *α_s_* was set to 0.5 or 0.6. Therefore, this paper eliminated the enhanced images with these two parameters and only discussed the image enhancement results with low *α_s_* value(*α_s_* < 0.5). Moreover, the highest value and the second highest value of PSNR and IE were mainly concentrated in the results of *α_s_* =0.3 and *α_s_* =0.4, which indicates that under the above two parameter settings, the images had a good performance in terms of image information, contrast enhancement and detail preservation. Furthermore, for *α_s_* =0.4, both the IE and AG values were higher than those for *α_s_* =0.3, while the PSNR was slightly lower than the latter. Therefore, based on the qualitative evaluation results and the requirements of enhanced images in terms of clarity, information content, picture details and contrast, *α_s_* was finally set at 0.4 in this study.

### 3.2 Unstructured road extraction

In this section, unstructured road extraction was achieved by fusing two parts, including the segmented road region after removing green regions from the HSV space and road region based on improved gray factor.

#### 3.2.1 Road extraction based on color enhancement and HSV color space

HSV color space is composed of hue (H), saturation (S) and luminance (V) channels. As HSV color space is more consistent with human color perception, it has been widely used in multifield research based on machine vision, such as medicine (Singh, 2020), agriculture (Liao et al., 2022), and chemical industries (Safarik et al., 2019). Therefore, the HSV color space was used here to extract road regions.

**Figure 6:**
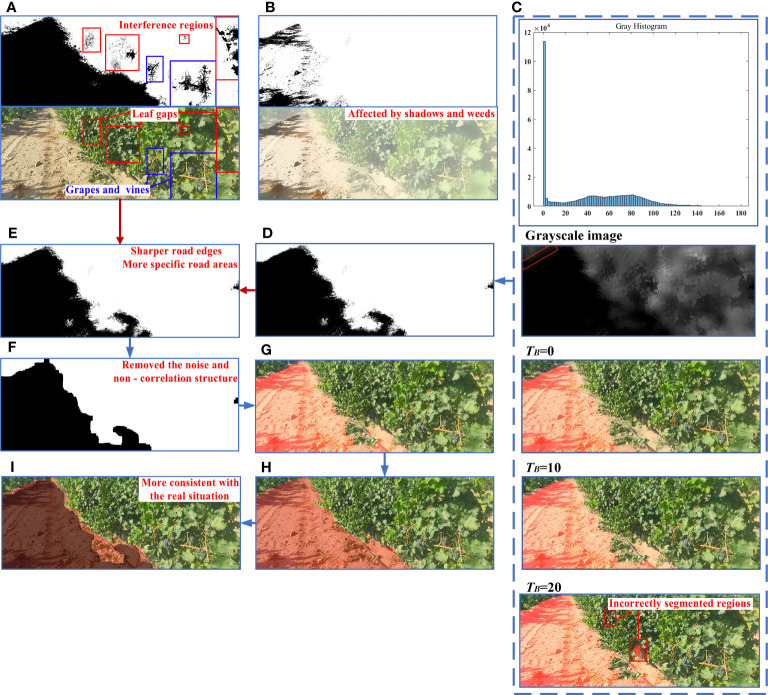
Process and results of road extraction. **(A)** Road extraction results in HSV space. **(B)** Road extraction results based on ExG Gray factor. **(C)** Road segmentation effect under different *T_B_*. **(D)** Road extraction results based on optimized grayscale factor. **(E)** Fused binary image. **(F)** Morphological processing result. **(G)** Final extraction result. **(H)** Manual image segmentation. **(I)** Results of comparison between proposed algorithm and real situation.

First, the enhanced and optimized RGB image was converted into an HSV image and the threshold range (H_min_, H_max_), (S_min_, S_max_), and (V_min_, V_max_) of each channel set to binarize the image. This completed the constraint and extraction of the green area, so as to distinguish the road area from the plant area (vines, weeds, and background trees). Based on Exploratory data analysis (EDA) and empirical values (Guo et al., 2013; Peng et al., 2013; Camizuli and Carranza, 2018), the HSV ranges were set at (35,77),(43,255),and (46,255), respectively (**Figure 6A**). As can be seen from the image, although the road extraction was relatively complete, the main constraint in the HSV space was the green region, such that there were still interference regions due to grapes and their vines, leaf gaps, and other factors in extraction results.

#### 3.2.2 Road extraction based on gray factor optimization

Taking advantage of the significant color difference between different objects in the image, numerous researchers have realized object segmentation by examining different gray weights, such as excessive red plant index (ExR, Meyer et al., 1999), excessive green index (ExG, Woebbecke et al., 1995), and normalized difference index (NDI, Woebbecke et al., 1993). The preprocessed image mainly contained four areas: grape vines regions, soil areas, background, and shadow areas. Therefore, through manual segmentation of the above regions and obtaining the average values of R, G, and B in different regions, the gray factor was improved by a heuristic method based on the excessive green index (ExG). The optimized gray factor and its binarized image acquisition formula were expressed in Equations 8 and 9 as


(8) gray(x,y)=1.84G(x,y)−B(x,y)−R(x,y)

(9) $$f(x,y)=\{\begin{array}{c} 0\cdots \cdots \cdots 1.84G(x,y)-B(x,y)-R(x,y)\leq T_{B}\\ 255\cdots \cdots \cdots 1.84G(x,y)-B(x,y)-R(x,y)>T_{B} \end{array}$$

where gray(x,y) is the optimized gray level factor, f(x,y) the binarized image, and G(x,y), B(x,y), and R(x,y) as the green, blue, and red components of the color range, respectively. And *T_B_* is the binarization threshold.

Based on the optimized grayscale factor, the grayscale image and the grayscale histogram of the enhanced image after the S-component were plotted in **Figure 6C**. As can be seen from the gray histogram, most pixels in the image had a gray value of 0, corresponding to the majority of black road areas in the gray map. However, as shown by the red area in the grayscale image, a few pixels in the road area had gray values that were not zero. Therefore, the rationality of the binarization threshold *T_B_* directly affected the integrity of the road segmentation. To determine the optimal binarization threshold, a comparative experiment was conducted in this paper, using the threshold value *T_B_* as the independent variable and the road segmentation result as the dependent variable. The initial value of the binarization threshold was set to 0, and different binarization thresholds were used to segment the road. The threshold of binarization was increased by 10 for each group until the segmentation result incorrectly included the vine area on the side of the road.

When *T_B_* = 0, the segmentation result indicated a significantly smaller road area than the actual road. With *T_B_* set at 10, the vast majority of road area was accurately extracted from the segmentation results. However, when *T_B_* was increased to 20, while the extracted road area was more comprehensive, there were numerous incorrectly extracted sections. Consequently, for this article, *T_B_* was established at 10, the road extraction results were shown in **Figure 6D**.

The extraction method of unoptimized gray factor based on ExG was found to be affected by shadows and weeds, resulting in a large number of noise points and holes in the treatment results, and only extracted a small number of road regions (**Figure 6B**). Thus, the extracted area was significantly smaller than the real value. On the other hand, the improved gray factor method exhibited superior segmentation results for the grapevine area on the road and its surroundings, showing great advantages in the accuracy and integrity of road segmentation (as depicted in **Figure 6D**). The above results indicated that compared with the unimproved gray factor, the improved gray factor method was more adaptable to unfavorable environmental conditions such as shadows and lighting in the field.

#### 3.2.3 Binary images fusion and morphological processing

By fusing the above two binarized images in **Figures 6A, D**, most of the disturbances (**Figure 6E**) were eliminated and road edges constrained. The fused results were more consistent with the real situation.

However, there were various tiny noises and irregularly-shaped edges in the fused binary image. Therefore, morphological processing was performed on fused binary images to remove non-correlated structures (**Figures 6F, G**).

The road edge extracted by this algorithm was found to be in line with the trend of the real road and fundamentally eliminated the vine area on the side of the road (**Figure 6I**). This reduced the interference of light, shadow, weeds, and dead branches to road extraction, with high extraction integrity and good comprehensive performance.

#### 3.2.4 Performance evaluation indexes

In this study, the number of ROI image pixels (NRP) and the ratio between the wrongly extracted pixels and the number of ROI image pixels (RBP) were used as evaluation indices for verifying the performance of the road extraction algorithm. And the calculation equations of this evaluation index expressed in Equation 10 as


(10) $$RBP=\frac{NWP}{NRP}\times 100\%$$

where NWP is the number of wrongly extracted pixels by the algorithm.

### 3.3 Roadside fruit detection based on YOLOv7

#### 3.3.1 Characteristics of the YOLOv7 network structure

**Figure 7:**
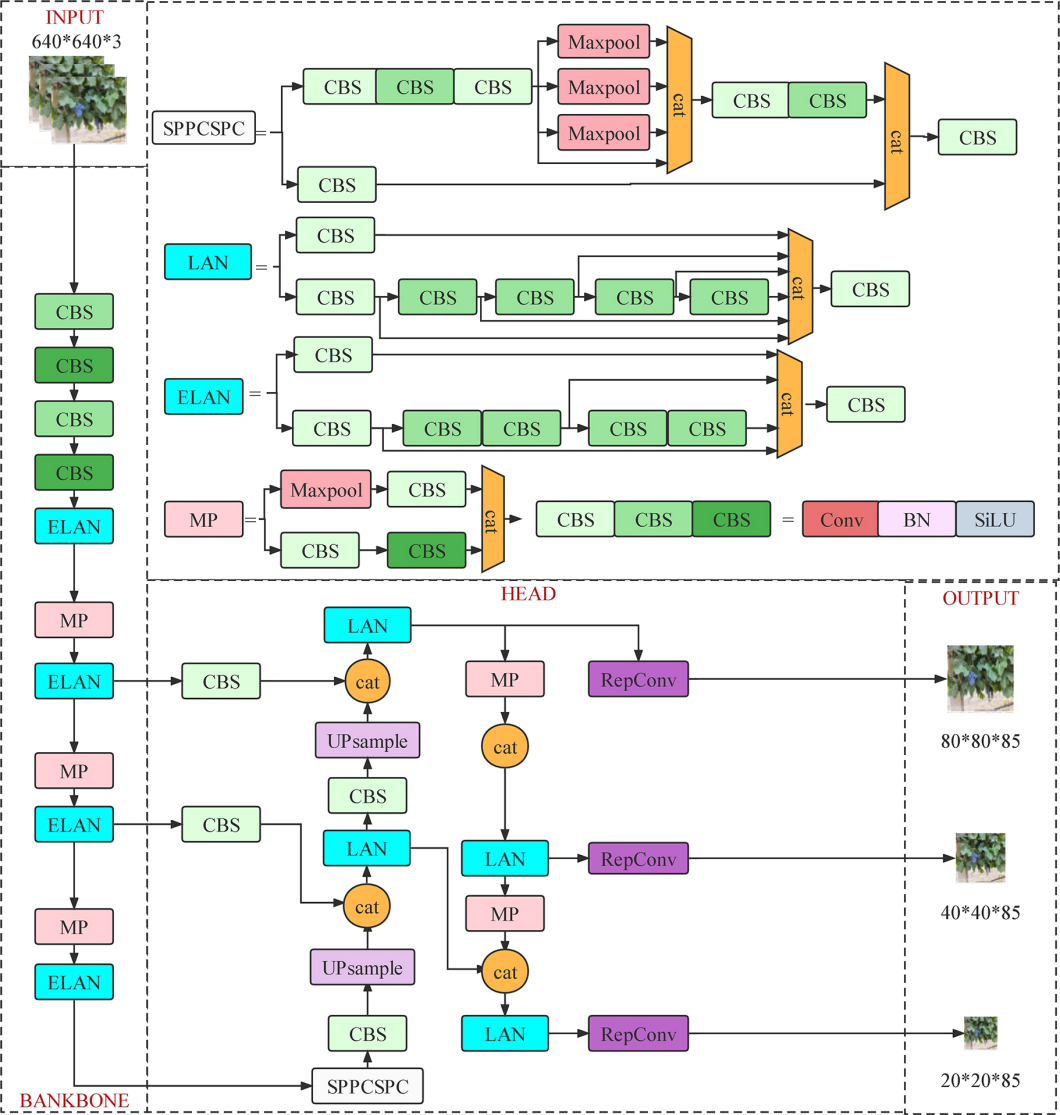
Network structure of YOLOv7.

As the latest version of the YOLO series (Wang C. et al., 2022), YOLOv7 has improved the existing model in many ways. First, it offers extended efficient layer aggregation networks (E-ELAN) based on ELAN structure, which can guide different computing blocks to learn more different features and enhance the learning ability of the model on the basis of maintaining the original gradient path. Then, a compound model scaling method based on the cascade model has been proposed to ensure the initial characteristics and optimal structure of the model, which efficiently utilizes parameters and computation. Meanwhile, several trainable bag-of-freebies methods have been designed for real-time object detection, which significantly improves detection accuracy without increasing inference cost. Based on the above improvements, YOLOv7 shows great advantages in terms of speed and accuracy over other detection algorithms. Its network architecture is shown in **Figure 7**.

Based on the performance advantages of the YOLOv7 and YOLOv5 models, both models were adopted in this research to detect roadside fruits. The results were compared to identify the roadside grape detection model that is better suited for large-field environments. The selected model’s feasibility and detection performance were then further verified for roadside fruit recognition.

#### 3.3.2 Network training and parameter optimization

The experiment was conducted on a Windows 10 operating system, with the Python framework, YOLOv7, and YOLOv5 environments built in the Anaconda environment. The program was written in Python 3.9 and CUDA Ver. 11.7. In terms of hardware, the processor is an Intel (R) Core (R) i5-1240F CPU@2.5 GHz, the dominant frequency is 2.5 GHz, internal storage 32.0 GB, and graphics card an NVIDIA GeForce RTX 3060.

Due to the complex orchard environment, directly applying the default parameters of YOLO model to the roadside fruit recognition model results in poor detection results. To adapt to fruit recognition in complex field scenarios, the learning rate parameter of the YOLO model was chosen as described in this study. The initial value of the learning rate was set to 0.01 and the model was trained with different learning rates. The learning rate of each group was reduced by 0.002, respectively, until the optimal parameters were detected and chosen. By comparison, it was found that when the learning rate was larger than 0.002, the loss curves for object detection in the results suffered from severe oscillations, poor convergence or nonconvergence. Thus, the learning rate of the wine grape orchard recognition model was set to 0.002.

The training and verification sets were input into the network for training, with a batch size of 16 and 150 epochs, respectively (**Table 1**).

**Table 1:** YOLO basic parameters.

Parameters of model	Value
Input image resolution	640×640
Learning rate	0.002
Momentum	0.937
Optimizer weight decay	0.0005
Warmup momentum	0.8
Batch size	16
Training epochs	150

#### 3.3.3 Model evaluation

In this study, precision (P), recall (R), F1-score, and *mAP* were used as the evaluation indices of roadside fruit detection performance and the calculation equations of each evaluation index expressed in Equations 11–14 as:


(11) $$P=\frac{TP}{TP+FP}$$

(12) $$R=\frac{TP}{TP+FN}$$

(13) $$F1-score=\frac{2\times P\times R}{\left(P+R\right)}$$

(14) $$mAP=\frac{\sum_{1}^{c}AP(c)}{c}$$

where *TP*, *FP*, and *FN* correspond to true positives (there is a grape bunch in the image and the algorithm predicts it correctly), false positives (there are no grapes in the image, but the algorithm detects it), and false negatives (the algorithm failed to detect a bunch of grapes which are actually in the image), respectively, and *C* the number of detection classes. As only one kind of fruit was identified in this study, *C* = 1.

## 4 Experiments and discussion

By achieving synchronous recognition of road extraction and roadside fruit, this algorithm can considerably improve the ability of robots to perceive critical information in the orchard environments and lay the foundation for autonomous walking and picking decisions based on machine vision. Therefore, the performance of this algorithm was extremely critical for the robot’s picking rate, navigation path extraction accuracy, and reliability of the decision system in subsequent researches. At the same time, this study served as a reference for other research in the same field.

In this section, the performance of image enhancement, road extraction, roadside fruit recognition, and overall fusion algorithm were verified and discussed.

### 4.1 Road extraction effects and ablation tests

#### 4.1.1 Road extraction results and analysis

To validate the image segmentation effect of the proposed road extraction algorithm, the results obtained by fused segmentation were compared with those obtained by the conventional color image method. This study adopted two traditional algorithms: a method based on S component and Otsu and another based on the Excess Green index (ExG) and Otsu. At the same time, 25 images with pavement shadows, strong illumination variations, and grapevines with different degrees of color were selected as test samples to verify the adaptability of the above algorithms to complex environments.

**Figure 8:**
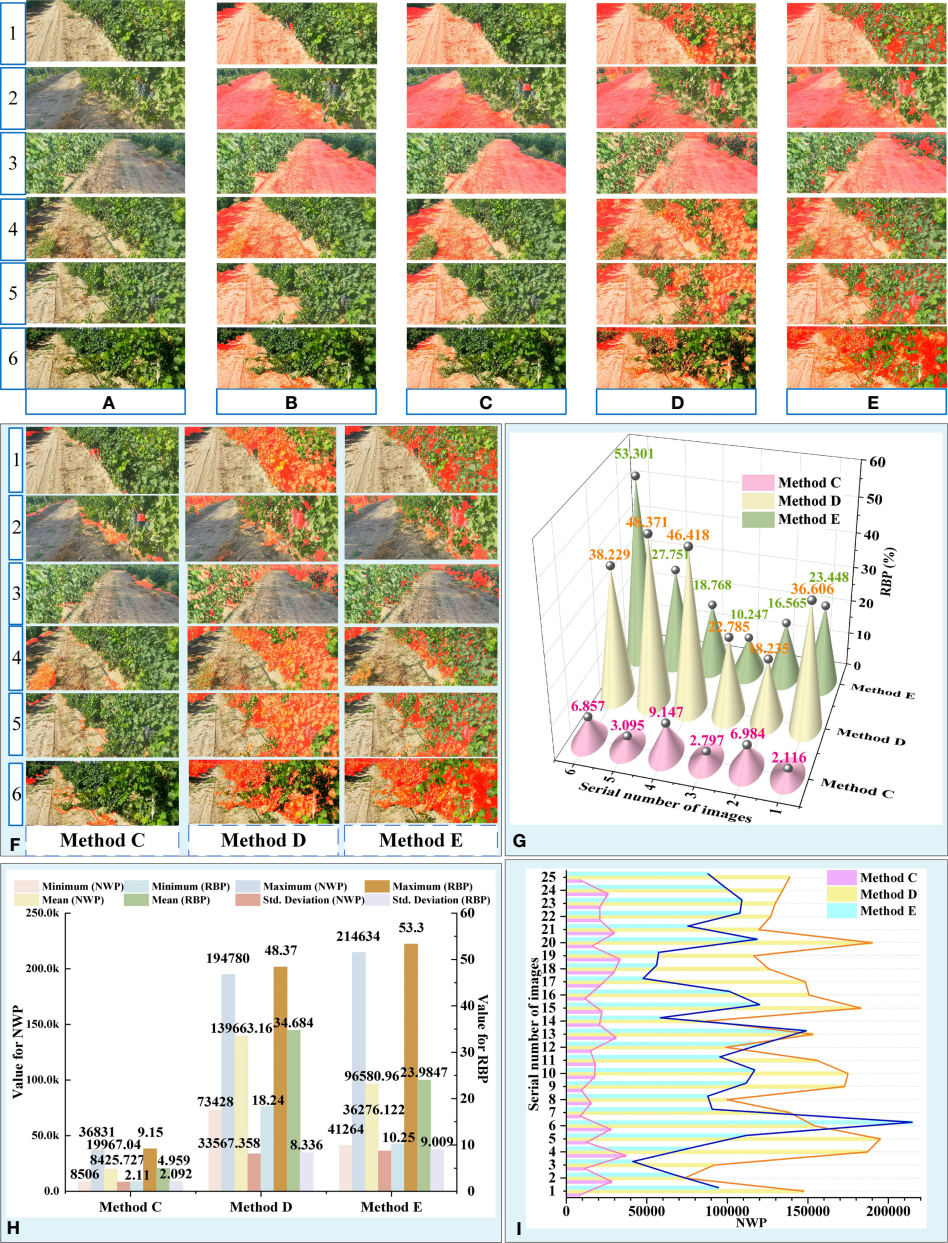
Results and analysis of different segmentation methods. **(A)** Original images. **(B)** Manual image segmentation. **(C)** Proposed algorithm. **(D)** Method based on S component and Otsu. **(E)** Method based on EXG and Otsu. **(F)** Error area results extracted by different methods. **(G)** RBP values of partial images obtained by different methods. **(H)** Descriptive Statistics for NWP and RBP. **(I)** NWP values of 25 images obtained by different methods.

The results of multiple sample images were compared, in which samples were original color images and other images obtained by segmentation methods. The comparative findings for partial sample images were illustrated in **Figures 8A–F**, while the comparative results for additional images could be found in the **Supplementary Material**. **Figures 1**–**3** depict the image samples with the lowest NWP value in the outcomes of Methods C, D, and E, while **Figures 4**–**6** depict the image samples with the highest NWP value in the outcomes of Methods C, D, and E, respectively.

For simplicity, the proposed algorithm was abbreviated as “Method C”, the method based on S component and Otsu was abbreviated as “Method D”, and the method based on EXG and Otsu was abbreviated as “Method E”.

In the qualitative evaluation, the quality of different segmentation methods was assessed based on the completeness of road segmentation and the distribution of error areas. Due to the complexity of the field orchard, the primary environmental factors that influence the precision of road segmentation outcomes include the grapevine area, shadowed road area (Li et al., 2018), roadside unevenly colored area, and high contrast between light and dark areas (Tang et al., 2023b). As depicted in **Figure 8A**, strong lighting caused the grapevine areas on the roadside to exhibit characteristics such as uneven light and shade and varying color tones. This led to a significant contrast between light and shade in the grapevine areas on both sides of the road. Additionally, different lighting angles resulted in distinct areas of shadow on the road surface, thereby increasing the complexity involved in segmenting orchard roads.

Observationally, it was found that the extraction results of methods D and E (**Figures 8D, E**) suffered from problems, such as the large area errors in identification. Although the extraction results were of great completeness, the results also contained a large number of incorrect regions (**Figure 8F**). By comprehensive comparison, the road obtained by the Method C was found to be the closest to the real situation and had the best segmentation effect among all considered methods.

To further analyze the adaptability of the above method to complex vineyard scenarios, the extraction results of the proposed algorithm were compared with real roads (**Figures 8F, G**). Based on **Figure 8F**, it can be observed that the error areas of methods D and E were primarily concentrated in the grapevine area on the side of the road.

Method D was found to be sensitive to changes in brightness, shade, and color uniformity of the grapevine region in the image, which resulted in changes in the error area of the segmentation result (**Figure 8D**). Due to the unpredictable and random nature of illumination in field environments, it was difficult to guarantee the accuracy and stability of the segmentation results achieved through method D.

The primary error source of method E was the grapevine area with strong contrast between light and shade, with the dark part of it being incorrectly identified as the road area. This greatly reduced the accuracy of the segmentation result. When the area of the dark region of the grapevine on the side of the road was small, the error rate of this algorithm decreased significantly. However, when faced with areas that had uneven colors on the side of the road, the error area of the segmentation result achieved through this method was significantly smaller than that of method D.

Conversely, Method C adapted to the aforementioned unfavorable factors, resulting in a smaller error in the segmented area, more stable road extraction performance, and the most reliable segmentation results among the three methods. Combined with the above analysis, the influence degree of unfavorable factors on the accuracy and reliability of the results obtained through different methods was comprehensively evaluated, as presented in **Table 2**.

**Table 2:** Analysis of the influence degree of adverse factors on algorithms and extraction results.

Degree of influence of adverse factors on algorithm accuracy					
Adverse environmental factors	Impact degree				
	Method C			Method D	Method E
Grapevine area	Minor			Severity	Severity
Shadowed road area	Minor			Minor	Minor
Roadside unevenly colored area	Minor			Severity	Medium
Strong contrast between light & dark	Minor			Severity	Severity
Methods	Descriptive Statistics for NWP				
	Minimum		Maximum	Mean	Std. Deviation
Method C	8506		36831	19967.040	8425.727
Method D	73428		194780	139663.16	33567.358
Method E	41264		214634	96580.960	36276.122
Pairwise Comparisons of Methods (NWP)					
*Sig*	Method C vs Method D			Method C vs Method E	Method D vs Method E
	<0.001			<0.001	0.008
Methods	Descriptive Statistics for RBP/%				
	Minimum		Maximum	Mean	Std. Deviation
Method C	2.11		9.15	4.959	2.092
Method D	18.24		48.37	34.684	8.336
Method E	10.25		53.30	23.9847	9.009
Pairwise Comparisons of Methods (RBP)					
*Sig*	Method C vs Method D			Method C vs Method E	Method D vs Method E
	<0.001			<0.001	0.008

To quantitatively evaluate the extraction performance of the above methods, NWP and RBP were taken as indices to achieve a road extraction performance evaluation of different algorithms, where NRP = 402,668 (**Table 2**; **Figures 8H, I**). To determine the differences in road extraction performance among the three methods, the non-parametric Kruskal-Wallis test was conducted across the three groups using SPSS software version 27 (IBM Corporation). The significance level was set at 0.05. The null hypothesis in this test is that there is no difference between the three methods in terms of the distribution of NWP and RBP. In fact, for this test, the *Sig* values less than 0.05 indicate a significant difference between the groups.

According to the descriptive statistical table of NWP, Method C exhibited a generally low overall level of NWP value (**Figure 8H**). Comparing the mean value of NWP across the three methods, it was found that the mean value of NWP for Method C accounted for only 14.3% and 20.67% of the mean value of NWP for Methods D and E, respectively. Furthermore, the maximum and minimum values of NWP for Method C were one order of magnitude smaller than those of Methods D and E. Additionally, the standard deviation of NWP value for Method C was significantly lower than that of Methods D and E, indicating that the road extraction performance of Method C was more stable in the face of variable field interference factors. This observation was also validated in **Figure 8I**, which illustrates that the NWP of Method C exhibits a relatively mild fluctuation in comparison to the other two methods. Moreover, the Kruskal-Wallis test results showed that the NWP values of Methods C and D (*Sig*<0.01), Methods C and E (*Sig*<0.01) and Methods D and E (*Sig* = 0.008)were statistically significant difference. Furthermore, it was confirmed that there were substantial differences in the accuracy of road extraction among the three methods.

Similar results were obtained from the descriptive statistical table of RBP. Method C demonstrated favorable outcomes in terms of the maximum, minimum, mean, and standard deviation of RBP. Thereinto, Method C had an RBP of no more than 9.15%, whereas Method D had an RBP of no more than 48.37%, and Method E had a notably high RBP of 53.30%. The above data suggested that the wrongly identified pixels in the road extraction results of Method C only constituted a small portion of the current image. Compared to the other two methods, Method C was found to deliver better segmentation results for road recognition in the field environment and exhibited greater adaptability to the complex environmental interference factors in the field orchard.

#### 4.1.2 Ablation test

**Figure 9:**
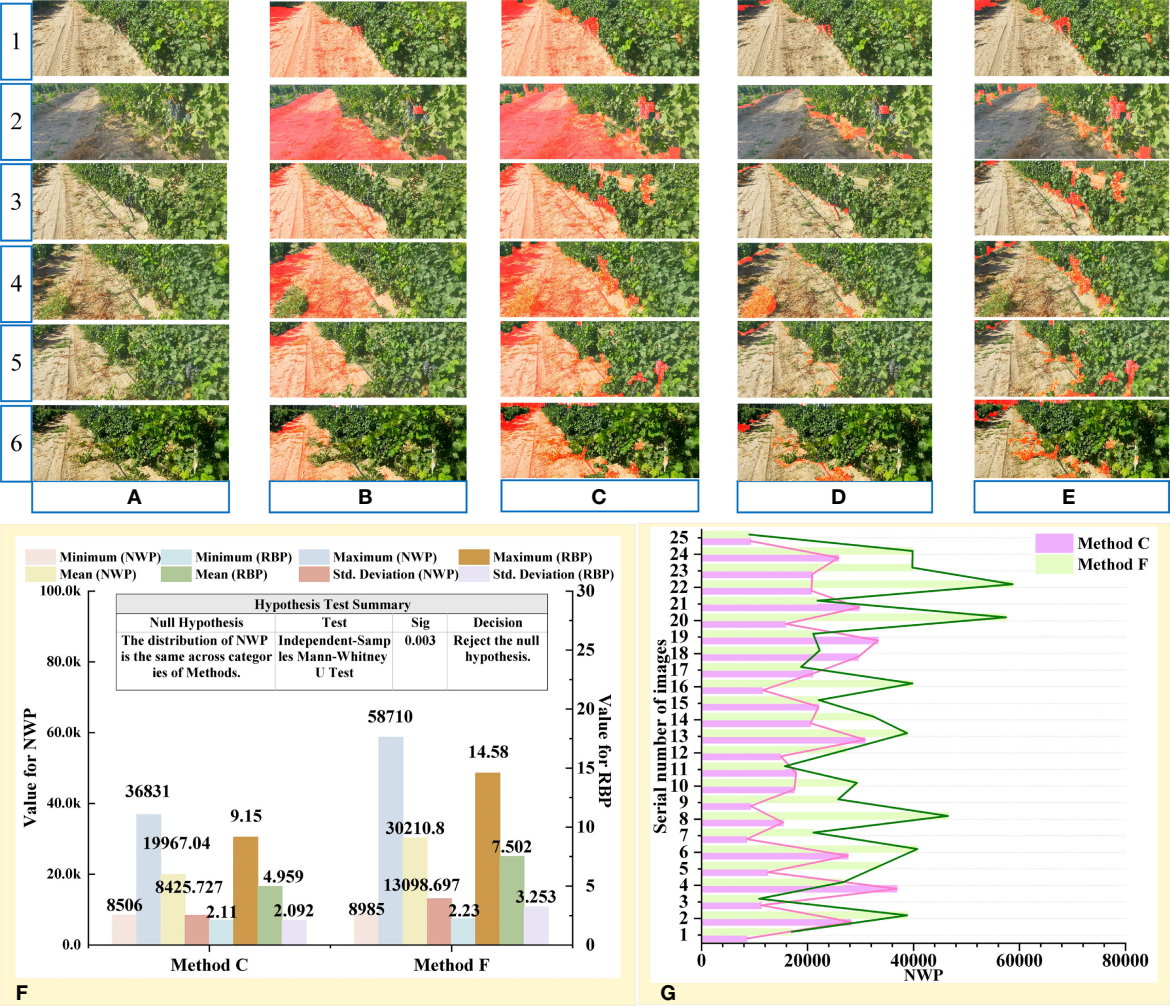
Ablation test results from the proposed preprocessing method. **(A)** Original images. **(B)** Proposed algorithm. **(C)** Proposed algorithm without preprocessing. **(D)** Error area results extracted by proposed algorithm. **(E)** Error area results extracted by proposed algorithm without preprocessing. **(F)** Descriptive Statistics and significance analysis result. **(G)** NWP values of 25 images obtained by methods C and F.

To verify the improvement of the image enhancement algorithm on the overall performance of the road extraction algorithm, an ablation experiment was conducted. The comparative findings for a selection of sample images were illustrated in **Figure 9**, while the comparative results for additional images could be found in the **Supplementary Material**. For simplicity, the proposed algorithm without preprocessing was abbreviated as “Method F”.

Ablation experiments were conducted on the proposed preprocessing method. The extraction results after pretreatment were shown in **Figure 9B** and the algorithm results without pretreatment were shown in **Figure 9C**. By comparing the two extraction results, the latter extraction results were found to contain a large number of error regions, such as dark grape vines area, grapes, and other objects on the roadside (**Figure 9E**). This phenomenon was confirmed by NWP descriptive statistics (**Figure 9F**).

Based on **Figures 9F, G**, it can be observed that the majority of segmentation results obtained using Method F had a higher NWP value compared to those obtained using Method C. However, a few image processing results showed an opposite result. The reason for this phenomenon can be attributed to the fact that after image preprocessing, the segmentation result of Method C had more stringent restrictions on green areas, resulting in the removal of a large area of weeds from the road in the segmentation results, thereby increasing the NWP value (4th row of **Figure 9C**).

After image preprocessing, the accuracy of the algorithm was significantly improved at the cost of a small amount of completeness, which reduced the impact of interference regions, such as road shadows, dark fruits, branches, leaves, and gaps in segmentation accuracy. Meanwhile, the Method C also suppressed the interference of noncurrent road areas on the extracted results and significantly reduced the number of misdetected pixels (3th row of **Figures 9C, D**).

Differences in road extraction performance between the above methods were determined using the non-parametric Mann-Whitney U test. The significance level was set at 0.05. The null hypothesis in this test is that there is no difference between the methods in terms of the distribution of NWP. And the *Sig* values less than 0.05 mean a significant difference between the groups. The Mann-Whitney U test result showed that the NWP values of Methods C and F (*Sig*= 0.03) were statistically significant difference (**Figure 9F**).

In conclusion, image preprocessing played a crucial role in enhancing the accuracy and reliability of road segmentation results.

### 4.2 Comparison between YOLOv5 and YOLOv7

Target location is an important task in target detection and is normally represented by the coordinate position of the bounding box. The models in this paper used CIoU (Lv et al., 2022) loss to calculate the boundary frame position loss, which was calculated as follows:


(15) $$L_{box}=1-IOU+\frac{\rho^{2}(A,B)}{c_{d}^{2}}+\alpha \upsilon$$

(16) $$\upsilon =\frac{4}{\pi^{2}}{\left(\arctan\frac{w^{g}}{h^{g}}-\arctan\frac{w^{p}}{h^{p}}\right)}^{2}$$

(17) $$\alpha =\frac{\upsilon }{\left(1+IOU\right)+\upsilon }$$

Where $\rho^{2}(A,B)$ is the Euclidean distance of the center points between predicted box and ground truth box, $c_{d}$ is the diagonal distance of the smallest rectangle containing predicted box and ground truth box, *α* is the weight function, and υ is the function that measures the consistency of the aspect ratio. $w^{g}$ and $h^{g}$ are the width and height of the ground truth box, while $w^{p}$ and $h^{p}$ are the width and height of the prediction box.

The confidence loss function is used to measure the difference between the confidence score predicted by the model and the actual label. In this paper, the confidence loss function was calculated using a binary cross-entropy loss function (BCELoss, Zhao et al., 2023), and its formula was as follows:


(18) $$L_{conf}=-\frac{1}{N}\sum_{n=1}^{N}\left[y_{n}\times \log x_{n}+\left(1-y_{n}\right)\times \log\left(1-x_{n}\right)\right]$$

Where $y_{n}$ denotes the true category, which generally takes the value of 0 or 1, $x_{n}$ denotes the prediction confidence or target probability obtained by the Sigmoid function, and N is the number of positive and negative samples.

**Figure 10:**
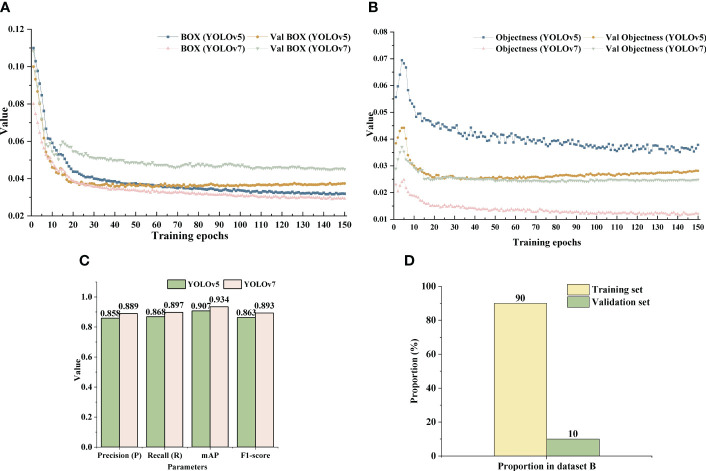
Loss curves and detection results of the two YOLO models. **(A)** Box loss value curve of YOLOv5 and YOLOv7 model. **(B)** Confidence loss function value curve of YOLOv7 model. **(C)** Detection results of YOLOv5 and YOLOv 7 on dataset B. **(D)** Training set and verification set introduction.

After training, the loss function value curves for the training and validation sets of the two YOLO models were obtained, including the loss values of the detection box and detection object (**Figures 10A, B**). In **Figure 10A**, “BOX” and “Val BOX” represented the box loss of the training set and validation set, respectively. In **Figure 10B**, “Objectness” and “Val Objectness” represented the confidence loss of the training set and validation set, respectively. As shown in **Figure 10A, B**, it can be observed that the change trend of the loss curves for both models was similar. In particular, it was observed that the values of box and object detection losses for the two YOLO models decreased sharply during training batches 0 to 20, after which the rate of decline slowed down. The sample distribution ratio of model training set and verification set is shown in **Figure 10D**. In addition, the box and the object detection loss values of the YOLOv7 algorithm on the training set were smaller than that of the YOLOv5 algorithm after 150 training epochs. The box detection loss value of YOLOv7 finally stabilized around 0.029 and object detection loss value eventually stabilized around 0.012.

In addition, although the loss value of box detection in the validation set was slightly higher than that of YOLOv5, the loss value of object detection in the validation set of YOLOv5 showed a trend of fluctuation and rise after 50 training batches. Meanwhile, the loss value of YOLOv7 algorithm decreased steadily and finally the loss value tended to stabilize around 0.0025.

Under the same dataset B, the performance indices of YOLOv7 were better than those of YOLOv5 (**Figure 10C**). The P, R, *mAP*, and F1-scores of YOLOv7 were 88.9, 89.7, 93.4, and 89.3%, respectively, which were 3.1, 2.9, 2.7, and 3% higher than from YOLOv5.

**Figure 11:**
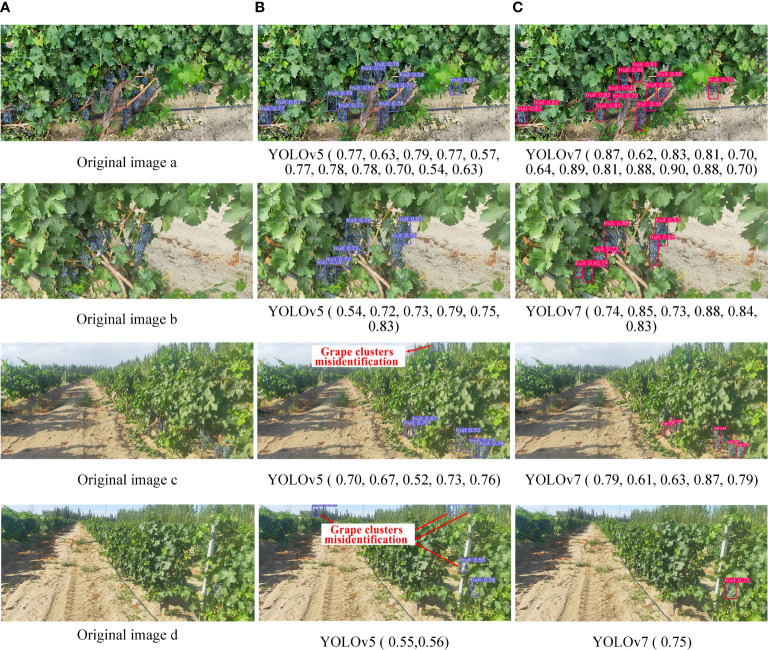
Comparison of partial detection results. **(A)** Original images. **(B)** Identification results of YOLOv5 model. **(C)** Identification results of YOLOv7 model.

Although the number of YOLOv7 targets detected in some images was less than that of YOLOv5, the overall accuracy of the former was higher than that of the latter (**Figures 11A–C**). Moreover, in global images, YOLOv5 showed the phenomenon of grape cluster misidentification (**Figure 11**, last row). Algorithm detection confidence was the main evaluation metric in this study. In summary, YOLOv7 was able to better perform the task of detecting clusters of grapes in orchards and, hence,YOLOv7 was used to identify grapes on the roadside.

The confidence level of grape clusters recognition results tested by YOLOv7 on dataset B was mostly above 0.8, while it was mostly above 0.5 on dataset A. There were two reasons for this phenomenon. The first was that the grape clusters were smaller on dataset A than those in the training set and the second that dataset A contained a large number of backgrounds, such as sky, trees, and roads, and the overall complexity of the image far greater than that of the training set.

### 4.3 Recognition effects of the synchronous detection algorithm

**Figure 12:**
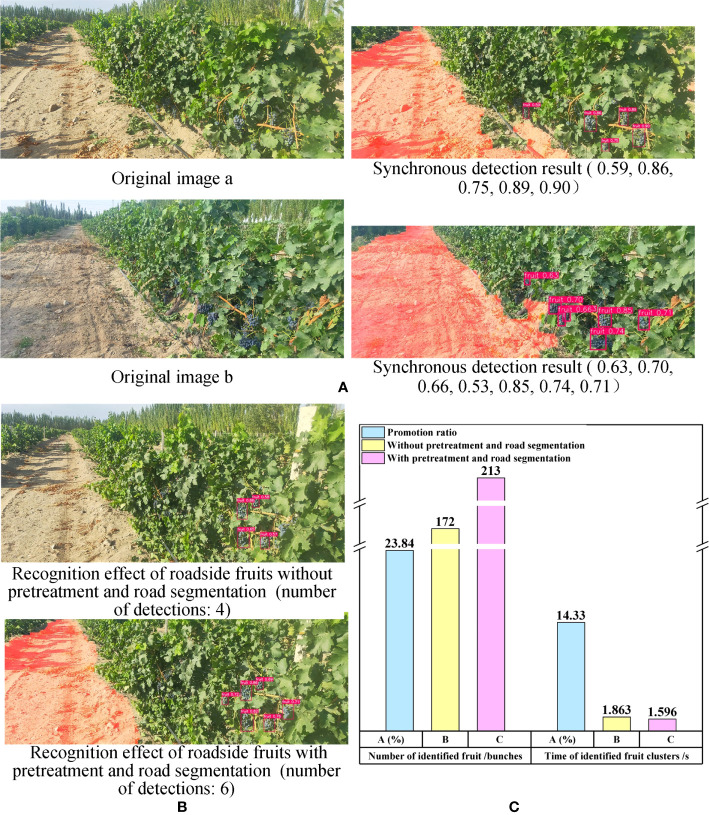
Recognition results of overall algorithm and comparison results between proposed algorithm and single YOLOv7 model. **(A)** The overall synchronous detection algorithm recognition results. **(B)** Comparison results of roadside grape clusters identification results between proposed synchronous detection algorithm and single YOLOv7 model. **(C)** Performance comparison between overall synchronous detection algorithm and the single YOLOv7 model.

Furthermore, in order to evaluate the overall detection performance of the synchronous detection algorithm proposed in this paper (**Figure 4**), simultaneous recognition of the road and roadside fruit was conducted (**Figure 12A**).

The results demonstrate that the algorithm was able to effectively segment the road area despite the complex outdoor environment, and accurately recognize the grapes on the side of the road. This provides valuable information for the intelligent decision-making and control of the robot during subsequent walking and fruit picking operations, and enhances the robot’s ability to identify crucial targets within a complex environment.

Furthermore, the synchronous recognition algorithm demonstrated better effectiveness in roadside grape recognition. To validate the positive impact of image preprocessing and road segmentation in the synchronous recognition algorithm on the recognition performance of road test grapes, the images with and without above aforementioned steps were identified using yolov7 model (**Figure 12B**). The results revealed that, under identical circumstances, the former approach detected more clusters of grapes on the road side.

To further demonstrate the superiority of the proposed synchronous recognition algorithm in roadside grape detection, 66 images from dataset B were used to detect grape clusters. The number of recognized fruits, recognition time and the promotion ratio ( $P_{r}$ ) were taken as evaluation parameters. The $P_{r}$ was calculated by the following formula.


(19) $$P_{r}=\frac{V_{w}-V_{n}}{V_{n}}$$

Here, $V_{w}$ represents the evaluation parameters obtained through image calculation based on image preprocessing and road segmentation, while $V_{n}$ represents the evaluation parameters obtained without image preprocessing and road segmentation.

The number of recognized grape clusters in the former was 41 more than that in the latter, representing a 23.84% increase. Additionally, the recognition speed of the former was 0.267 seconds faster than that of the latter, resulting in a speed increase of 14.33%. The results indicated that the images with pre-processing and road segmentation were able to identify more grape clusters and at a faster detection speed compared to the images without pre-processing and road segmentation (refer to **Figure 12C**). This finding provided evidence that the synchronous recognition algorithm proposed in this paper outperforms using YOLOv7 alone for identifying roadside grapes under the same scenario.

The reasons for the above phenomena were as follows: First, due to the extraction and preprocessing of the ROI in the overall algorithm, a large number of backgrounds, such as sky and trees, were eliminated, which improved the proportion of grape cluster pixels in the whole image. In addition, after extracting the road in the image, the interference of the road area on fruit cluster recognition was reduced and grape features more pronounced, which was beneficial for detecting fruit clusters on the roadside.

### 4.4 Discussion

**Figure 13:**
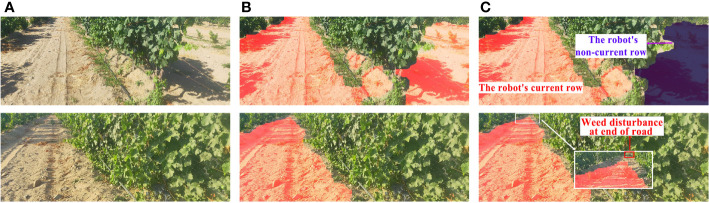
Adverse Conditions. **(A)** Original image. **(B)** Result of road extraction. **(C)** Analysis of adverse factors.

Although the unstructured road extraction and roadside fruit synchronous recognition algorithm proposed in this study had good performance, it also had some limitations (**Figures 13A–C**). First, it was difficult to distinguish the adhesive road areas between different rows during road extraction. For example, when the death of grape plants leads to a large area of vacancy on the road side, the road regions of images consisted of two parts: the road part of the robot’s current row and road part of the non-current row (**Figure 13C**). In this case, it was difficult for the proposed algorithm to distinguish the correct region from the wrong one. At the same time, when there were a large area of weeds near the end of the road with a width of more than 1/2 of the width of the road, the completeness of the extracted results was reduced. Future research will consider optimization algorithms and add constraints to improve result accuracy.

In addition, in the process of roadside fruit string identification, there was still a situation of missing grape-cluster detection. Future research will further optimize and improve the network structure for the problems of missing fruit string detection and low confidence of some detection target results.

## 5 Conclusions

In this study, an algorithm for unstructured road extraction and roadside fruit synchronous recognition in a complex orchard environment was developed to address the above issues. The main conclusions could be obtained as follows:

(1) An unstructured road extraction and roadside fruit synchronous recognition framework was constructed for achieving simultaneous road extraction and roadside fruit detection, which effectively improved the ability of fruit picking robots to extract key information from the picking environment. The algorithm also provided information for decision-making and reasoning of collaborative behavior of key parts of the robot, which improved the adaptability of the robot to randomly distributed fruit.
(2) Based on the analysis of the orchard images, an image enhancement preprocessing method was proposed to reduce the interference of road shadows, dark fruits, branches, and leaves as well as gaps in segmentation results. The method also suppressed the influence of noncurrent road areas on extraction results to a certain extent, which improved result accuracy and reliability.
(3) By enhancing the color channel and optimizing the grayscale factor, the dual spatial fusion road extraction was achieved. Experimental results showed that, compared with the extraction method based on S component and Otsu and extraction method based on EXG and Otsu, the proposed algorithm showed greater adaptability to adverse conditions, such as uneven illumination and road shadows under the background of complex orchards. The proposed road extraction algorithm also largely avoided the problems of missing extraction of real road areas and identification of large area errors, which had the best segmentation effect.
(4) The YOLOv7 and YOLOv5 algorithms, optimized with grape cluster target data, were used to identify roadside grape clusters. The optimized YOLOv7 model achieved a precision of 88.9%, recall of 89.7%, mAP of 93.4%, and F1-score of 89.3%, all of which were higher than those obtained from the YOLOv5 model. Based on this comparison, the YOLOv7 with optimized parameters was found to be more suitable for roadside grape recognition in wide-field views.
(5) The proposed fusion algorithm took the road extraction results as input and then identified fruit strings on the road side. The performance of the proposed fusion algorithm was superior to only using the YOLOv7 model. Compared with the single YOLOv7 model, the number of grape string detections and detection speed of the fusion algorithm were increased by 23.84% and 14.33%.

Although the new algorithm has achieved satisfactory results, there remains some room for progress. First, due to the similarity between different lines of the roads, the algorithm in this case had difficulty in segmenting the cohesive road area between different lines. At the same time, the completeness of the extraction results was reduced when there were a large area of weeds with a width ratio of 1/2 near the end of the road.

Future work will focus on network structure optimization to improve the accuracy and speed of road extraction and roadside fruit detection algorithms. Constraints between road zones will also been studied to enable the identification and segmentation of road zones between different lines. Furthermore, environment-aware robot behavioral decision control systems will be developed to enable collaborative decision planning and response control of picking and walking operations in complex orchard environments.

## Data availability statement

The original contributions presented in the study are included in the article/**Supplementary Material**. Further inquiries can be directed to the corresponding authors.

## Author contributions

XJZ designed the experiments. WT and ZY carried out the experiments. XZZ designed the study, analyzed the data, and wrote the manuscript. HM and XL supervised and revised the manuscript. All authors contributed to the article and approved the submitted version.
