# Supplementary material for: Unstructured road extraction and roadside fruit recognition in grape orchards based on a synchronous detection algorithm
Source: Front Plant Sci. 2023 Jun 2;14:1103276. doi: 10.3389/fpls.2023.1103276 (PMC10272741; doi:10.3389/fpls.2023.1103276)
Supplement: Supplementary file 1 [file DataSheet_1.docx]

Supplementary Material

# Supplementary Data

We uploaded a ZIP file named "Training results for YOLOv5 and YOLOv7", which corresponds to the data in Figure 9 of the article

# Supplementary Figures and Tables

## Supplementary Tables

**Supplementary** Tables 1 Comparison between different road extraction methods

| Road extraction mode | Scope of application | Advantages | Disadvantages |
| --- | --- | --- | --- |
| Segmentation method based on machine learning | Remote sensing satellite images based on structured or unstructured scenarios | This approach is able to obtain a large amount of data and has the capability to cover a large area.​ | This method is easy to lose details and is not suitable for the working environment of fruit picking robots |
|  | Stereo camera images based on structured or unstructured scenes | ​This approach allows access to a large amount of detailed information, which can help in the field operation of the device. | This method requires specific network training and a large number of training sets |
| Traditional algorithm based on image feature | Stereo camera images based on structured or unstructured scenes | This method does not rely on large sample data and model training | This method only focus on road extraction and ignore the distribution of fruits along the road, which leads to the serious problem that picking robots are not robust enough to adapt to the changing orchard environments |

## Supplementary Figures


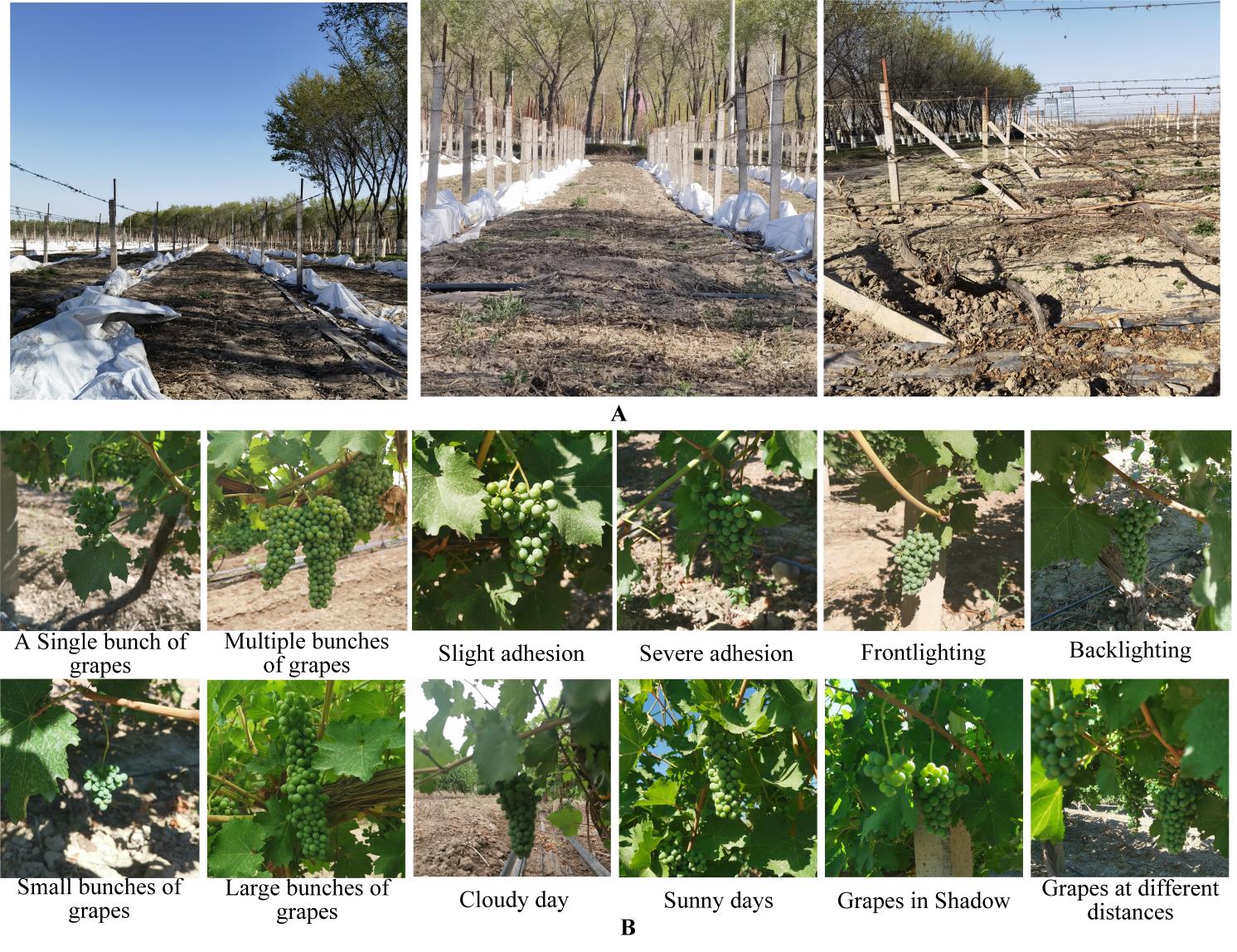


**Supplementary Figure 1.**  Natural images of vineyards and wine grape clusters at Changyu Babao Baron Winery in Shihezi. (A) Natural images of vineyards. (B) Natural images of wine grape clusters.


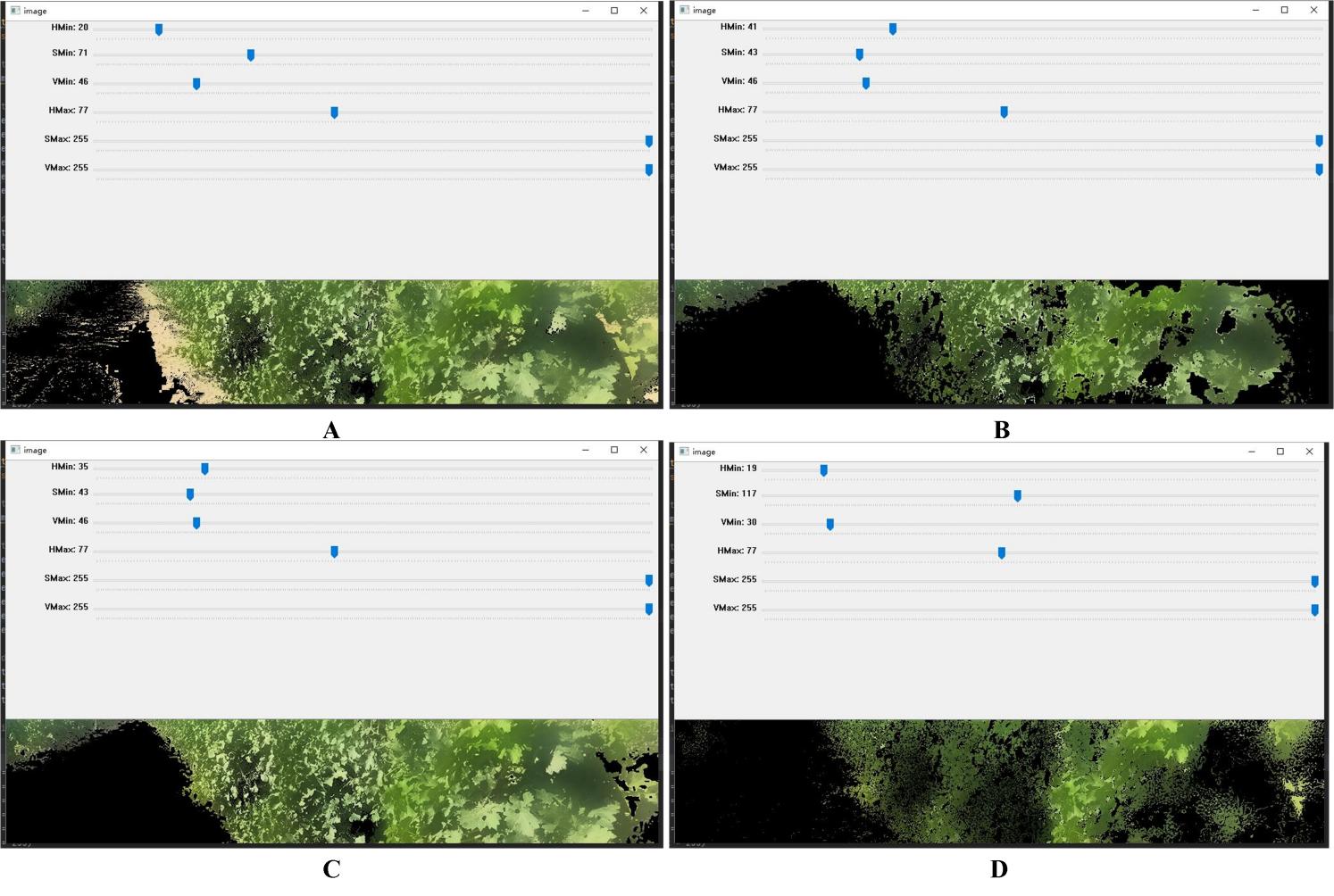


**Supplementary Figure 2.** Image segmentation effect under different HSV parameter range Settings. (A) (Hmin, Hmax), (Smin, Smax), and (Vmin, Vmax)=(20, 77), (71, 255), (46, 255). (B) (Hmin, Hmax), (Smin, Smax), (Vmin, Vmax)=(41, 77), (43, 255), (46, 255). (C) (Hmin, Hmax), (Smin, Smax), (Vmin, Vmax)=(35,77),(43,255) , (46,255). (D) (Hmin, Hmax), (Smin, Smax), (Vmin, Vmax)=(19,77),(117,255) , (30,255).


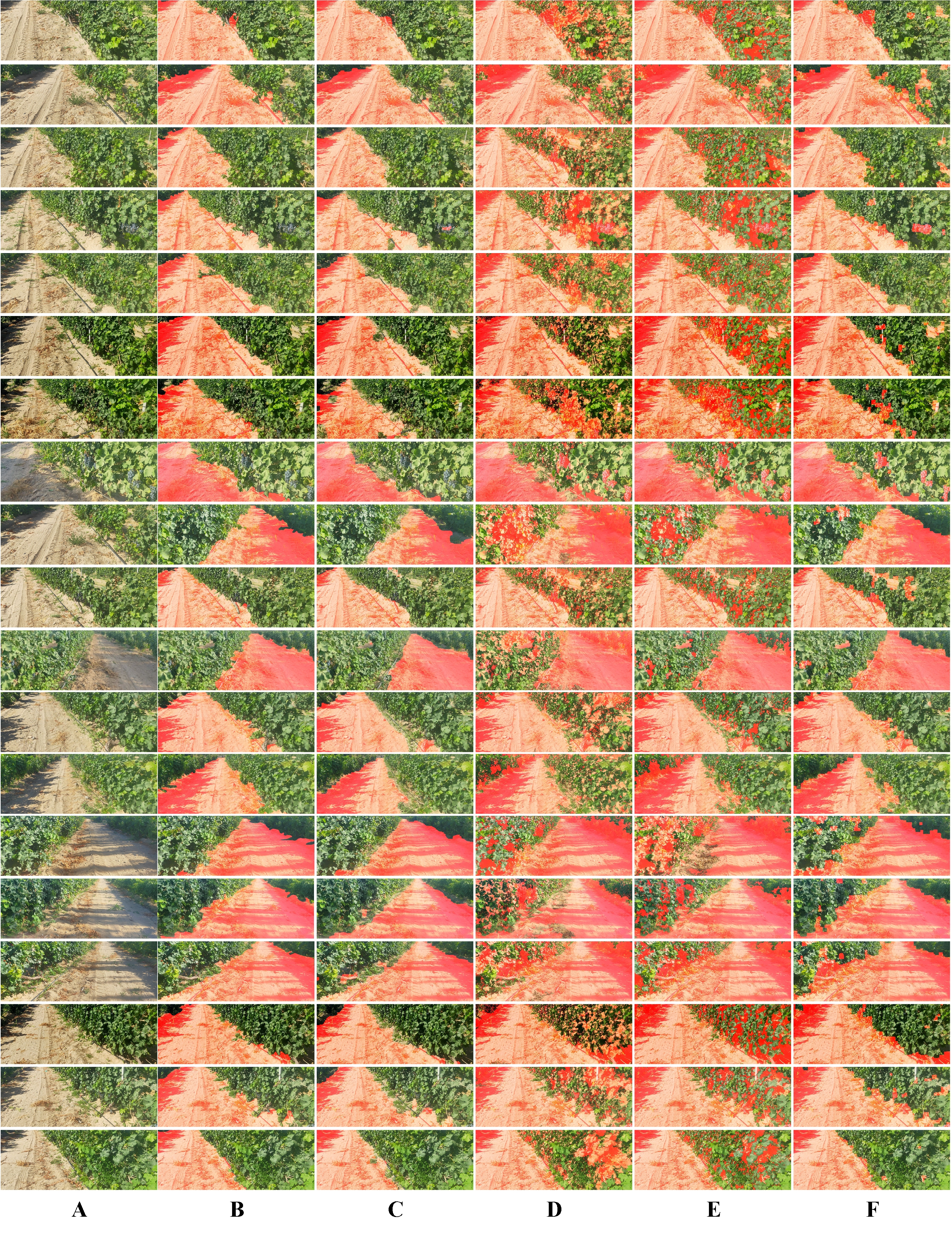


**Supplementary Figure 3.** The results and analysis of different segmentation methods for the remaining 21 images. (A) Original images. (B) Manual image segmentation. (C) Proposed algorithm. (D) Method based on S component and Otsu. (E) Method based on EXG and Otsu. (E) Proposed algorithm without preprocessing.
